# A novel epigenetic signature for overall survival prediction in patients with breast cancer

**DOI:** 10.1186/s12967-019-2126-6

**Published:** 2019-11-20

**Authors:** Xuanwen Bao, Natasa Anastasov, Yanfang Wang, Michael Rosemann

**Affiliations:** 1 Institute of Radiation Biology, Helmholtz Center Munich, German Research Center for Environmental Health, 85764 Neuherberg, Germany; 2 Technical University Munich (TUM), 80333 Munich, Germany; 3 Department of Pharmacy, Pharmaceutical Biotechnology, Center of Nanoscience (CeNS), Ludwig-Maximilians-Universität München (LMU), 80539 Munich, Germany

**Keywords:** Breast cancer, Mammary carcinoma, Epigenetics, Molecular marker, Response, Prognosis, Molecular signature, Individualized therapy

## Abstract

**Background:**

Breast cancer is the most common malignancy in female patients worldwide. Because of its heterogeneity in terms of prognosis and therapeutic response, biomarkers with the potential to predict survival or assist in making treatment decisions in breast cancer patients are essential for an individualised therapy. Epigenetic alterations in the genome of the cancer cells, such as changes in DNA methylation pattern, could be a novel marker with an important role in the initiation and progression of breast cancer.

**Method:**

DNA methylation and RNA-seq datasets from The Cancer Genome Atlas (TCGA) were analysed using the Least Absolute Shrinkage and Selection Operator (LASSO) Cox model. Applying gene ontology (GO) and single sample gene set enrichment analysis (ssGSEA) an epigenetic signature associated with the survival of breast cancer patients was constructed that yields the best discrimination between tumour and normal breast tissue. A predictive nomogram was built for the optimal strategy to distinguish between high- and low-risk cases.

**Results:**

The combination of mRNA-expression and of DNA methylation datasets yielded a 13-gene epigenetic signature that identified subset of breast cancer patients with low overall survival. This high-risk group of tumor cases was marked by upregulation of known cancer-related pathways (e.g. mTOR signalling). Subgroup analysis indicated that this epigenetic signature could distinguish high and low-risk patients also in different molecular or histological tumour subtypes (by Her2-, EGFR- or ER expression or different tumour grades). Using Gene Expression Omnibus (GEO) the 13-gene signature was confirmed in four external breast cancer cohorts.

**Conclusion:**

An epigenetic signature was discovered that effectively stratifies breast cancer patients into low and high-risk groups. Since its efficiency appears independent of other known classifiers (such as staging, histology, metastasis status, receptor status), it has a high potential to further improve likely individualised therapy in breast cancer.

## Background

Breast cancer is the most common tumour in woman, but represents a heterogeneous disease in terms of clinical prognosis and therapeutic response. Parts of the clinical heterogeneity can be linked to distinct molecular subtypes by gene expression profiles [1, 2]. Depending on the mutational and growth factor receptor status, a targeted chemotherapy had recently help to improve overall survival. DNA mutation and copy number changes are robust markers for molecular subtypes and they show little variation throughout therapy. However, their predictive value for progression and response may be limited. RNA expression pattern usually exhibits much larger variations between individual patients, and can be directly related to the activity of important pathways in malignant cells. On the other hand, RNA expression values also show relative rapid and stochastic variations, that could hamper the identification of relevant pathways. Epigenetic changes of DNA methylation are semi-stable and less variable, but show large variations related to the activity of cellular pathways. Thus, the combination of epigenetic status and transcriptome would be helpful for predicting the tumour progression. Moreover, Changes in DNA methylation provides tumour cells with a high level of plasticity to quickly adapt to changes in physiology, metabolic restrictions or to cytotoxic stress during therapy [3–5]. Therefore it is reasonable to analyse the DNA methylation pattern in the tumour cells in order to find novel predictors for the survival or response of breast cancer patients [6, 7].

The availability of high throughput genomic assays such as DNA methylation-seq, ATAC-seq and RNA-seq, have opened the possibility for a comprehensive characterisation of all molecular alterations of cancer cells and, hence to find novel biomarkers with clinical and therapeutic value [1, 8–10]. To overcome the limited statistical power of single biomarkers, entire molecular signatures derived from high-content genome screens seem to offer better predictive values. Some studies already demonstrated the power of whole transcriptome (RNA-seq) datasets, alone or in combination with DNA methylation datasets to build gene-based or CpG site-based signatures [6, 11]. In the present study, we merged DNA methylation and RNA-seq datasets of breast cancer patients from the Cancer Genome Atlas (TCGA) in order to develop a novel epigenetic signature, capable of predicting the overall survival. The proposed epigenetic signature was validated in 4 external datasets from the GEO database (617 cases in total).

## Methods

### Sample selection and data processing

TCGA DNA methylation datasets, RNA-seq datasets and clinical datasets for 1248 patients with mammary carcinoma were downloaded from the UCSC Xena browser (https://xenabrowser.net/). GSE12093, GSE17907, GSE20685 and GSE86948 were download from the GEO database. The detailed information for the patients was in Additional files 1 and Table 1.

**Table 1:**
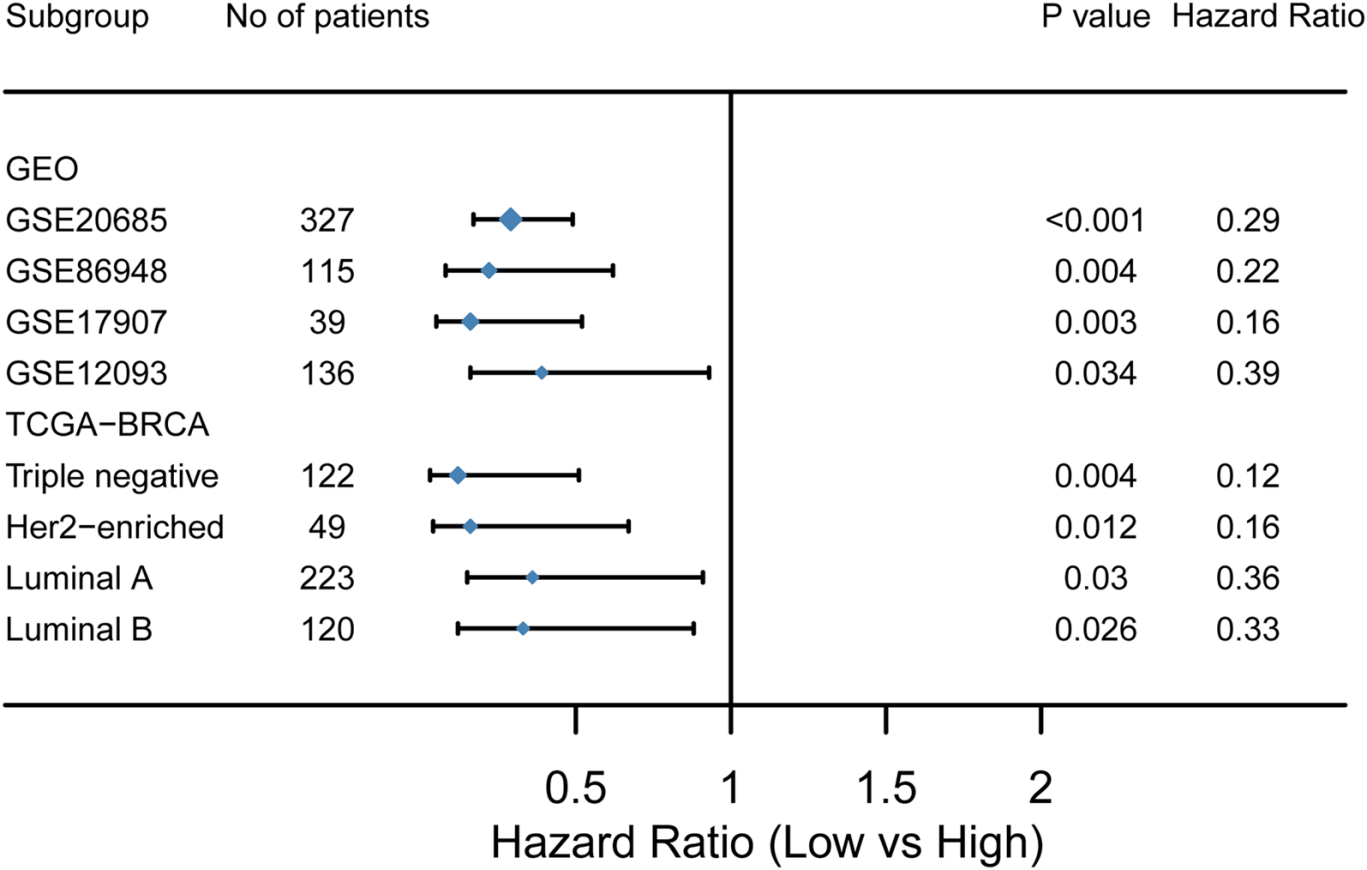
Detailed informations for each of the GEO cohorts and for the different breast cancer subtypes of the TCGA cohort are given, together with their calculated hazard ratio

### Differentially expressed genes (DEGs) analysis and differentially methylated genes (DMGs) analysis

Limma package was used to perform DEG analysis [12]. An empirical Bayesian approach was applied to estimate the gene expression changes using moderated t-tests. The DEGs were defined as genes with an adjusted p value of less than 0.05, and with an absolute of fold change greater than 2. The DMGs were defined as genes with an adjusted p value of less than 0.05, and an absolute β value (from HumanMethylation450 BeadChip) difference higher than 0.25. We could identify 306 genes that had overlapping changes in both DEGs and DMGs.

### LASSO regularisation and signature construction

LASSO (Least Absolute Shrinkage and Selection Operator) is a L1 regularisation method [13, 14]. An L1-norm is performed to penalise the weight of the features during regression model construction. The regularisation process forces the feature values to 0 and generate a sparse feature space. The correlation between mRNA expression and DNA methylation level of the overlapping genes from DEGs and DMGs were checked and the genes with absolute correlation greater than 0.3 in tumour tissues were used to build the prognostic model. 13 genes were selected for the construction of the signature and the coefficient for each was obtained through the penalizing process. A risk score formula was established as follows:

$$\mathop \sum \limits_{i} Coefficient\left( {mRNA_{i} } \right) \times Expression\left( {mRNA_{i} } \right)$$

Risk score = (0.321 * expression level of PCDHGA12) + (0.204 * expression level of HIF3A) + (0.061 * expression level of EZR) + (0.056 * expression level of PCDHGA3) + (0.044 * expression level of TPD52) + (− 0.011 * expression level of STAC2) + (− 0.012 * expression level of C2orf40) + (− 0.019 * expression level of KRT19) + (− 0.050 * expression level of NDRG2) + (− 0.054 * expression level of KCNH8) + (− 0.151 * expression level of CCND2) + (− 0.170 * expression level of SIAH2) + (− 0.186 * expression level of ITPRIPL1).

The nomogram was constructed by means of the rms package [15]. The ssGSEA was performed by package gsva [16].

### WGNCA for the transcriptome of breast tumour

A gene co-expression network was built by the Weighted Correlation Network Analysis (WGCNA) [10, 17]. Raising the co-expression similarity to a power *β* defined a weighted network adjacency [18]. By evaluating the correlations between the risk score of patients with breast cancer and the module memberships, it was possible to identify highly-correlated modules. The hub-gene (selected as gene significance greater than 0.4) in blue module was selected and underwent further analysis. Gene ontology (GO) and KEGG analysis were performed by clusterProfiler [19] and Metascape (metascape.org), respectively.

### Statistics

The correlation between mRNA expression level and DNA methylation level was analysed for every gene by Spearman correlation coefficient. Every dot represented the mean value of 80 samples in tumour tissues and the mean value of 10 samples in normal tissues in Fig. 5. The p value of the difference of gene expression in 4 molecular subtypes was calculated by ANOVA analysis. The p value and hazard ratio (HR) of survival analysis were calculated by COX regression. The code for analysing DEGs and risk scores were in Additional file 2.

## Results

Integrating TCGA breast cancer RNA-seq datasets with DNA methylation datasets according to the flowchart (Additional file 3: Figure S1) 306 genes were identified that form an overlapping cluster (up-regulated expressed genes overlap with hypomethylated genes and down-regulated expressed genes overlap with hypermethylated genes between tumour and normal tissues, respectively). Of these 306 genes, 95 genes had a significant correlation between the mRNA expression and DNA methylation values. LASSO Cox regression analysis build the prediction model with a 13-gene epigenetic signature as the best predictor for overall survival of breast cancer patients. ssGSEA was applied to identify the association between epigenetic signature and cancer-related hallmarks (e.g. MTORC1 signaling, G2M checkpoint). Using ssGSEA, WGCNA and downstream GO, KEGG analysis indicated that cell division, and cell cycle and related terms were closely linked to the signature. The nomogram which included the 13-gene epigenetic model and other clinicopathological factors exhibited high accuracy.

### Identification of differently expressed genes and differently methylated genes between tumour and normal tissues

**Fig. 1:**
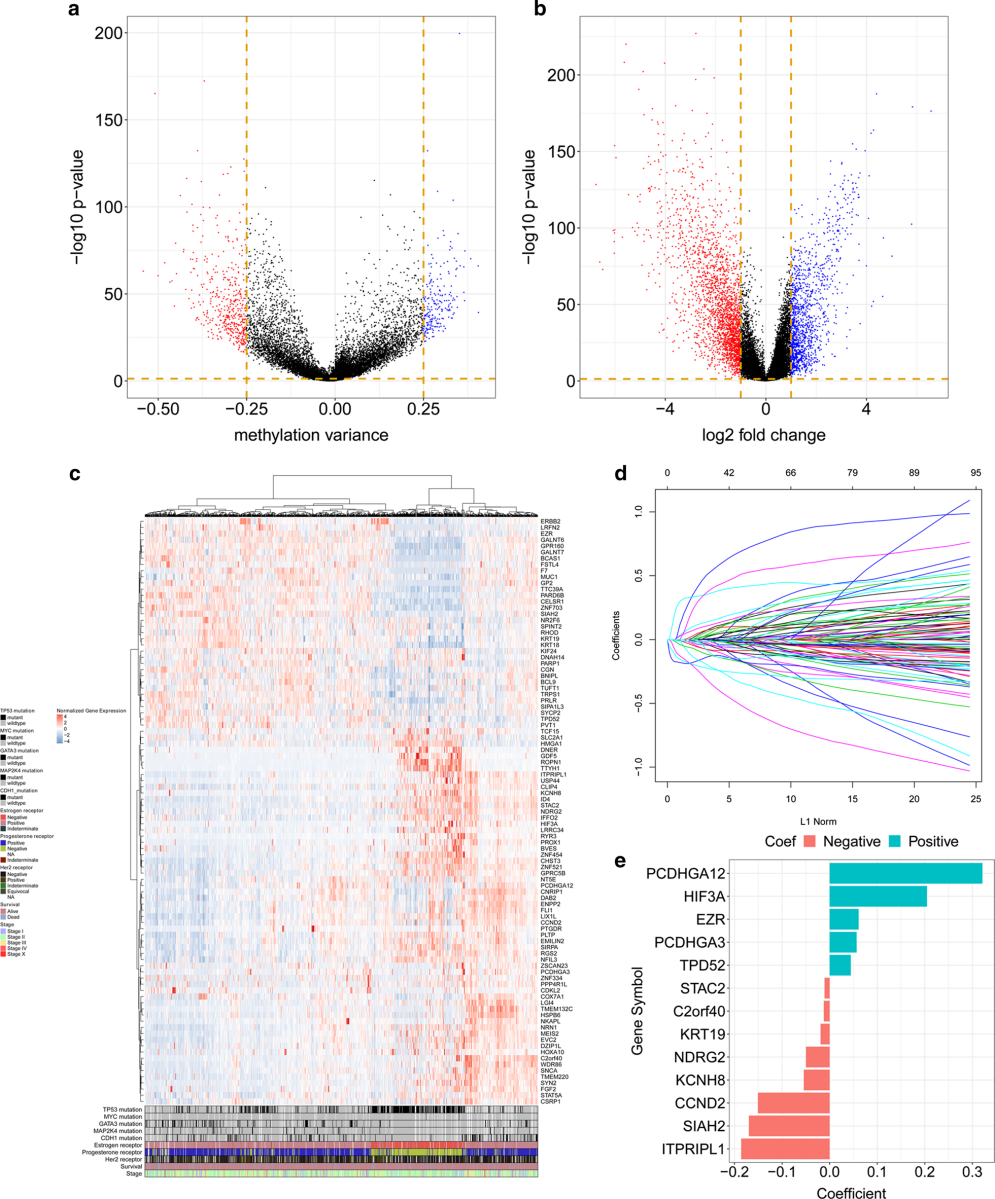
Construction of a prognostic epigenetic model in patients with breast cancer. **a** Volcano plot for DEGs in the tumour and normal tissues. **b** Volcano plot for DMGs in the tumour and normal tissues. **c** The expression of the DNA methylation-regulated genes shown by heatmap. **d** LASSO Cox regression model. **e** Coefficients distribution of the gene signature. *DEGs* differentially expressed genes, *DMGs* differentially methylated genes

The volcano plot (Fig. 1a) shows 3757 genes with a Ld2-FR of > 1, identified by the comparison of 1104 tumour samples and 114 normal samples. Analysing changes in the DNA methylation status, 225 were found to be hypomethylated and 446 genes hypermethylated in tumour tissues compared to normal tissue (Fig. 1b). Of those 671 genes with altered methylation status, 306 were also present among the 3757 genes with altered expression status. Of those co-regulated genes, 95 had DNA hypermethylation associated with a reduced mRNA expression level. The expression profile of the 95 genes showing negative correlation between methylation status and mRNA expression is shown in Fig. 1c together with the genomic characteristics and associated clinicopathological features.

### LASSO Cox regression identifying a 13-gene epigenetic signature

95 genes from above analysis constructed a gene-expression profile, and LASSO Cox model was applied to build the prognostic signature on the gene-expression profile. Cross-validation was carried out in 5 rounds to prevent overfitting (internal training sets and internal validation sets constructed randomly) (Fig. 1d).

The most powerful features (ITPRIPL1, SIAH2, KCNH8, KRT19, NDRG2, STAC2, TPD52, EZR, PCDHGA12, HIF3A, PCDHGA3, C2orf40, CCND2) were identified by the regularisation process of LASSO COX regression (Fig. 1e).

The ROC plots for identifying the tumour and normal tissues by expression level and methylation level of the 13 genes were shown in Additional file 4: Figure S2 and Additional file 5: Figure S3. The 13 genes showed high efficiency to differentiate between tumour and normal tissues in terms of both gene expression level and DNA methylation level.

### Overall survival prediction based on the epigenetic signature

**Fig. 2:**
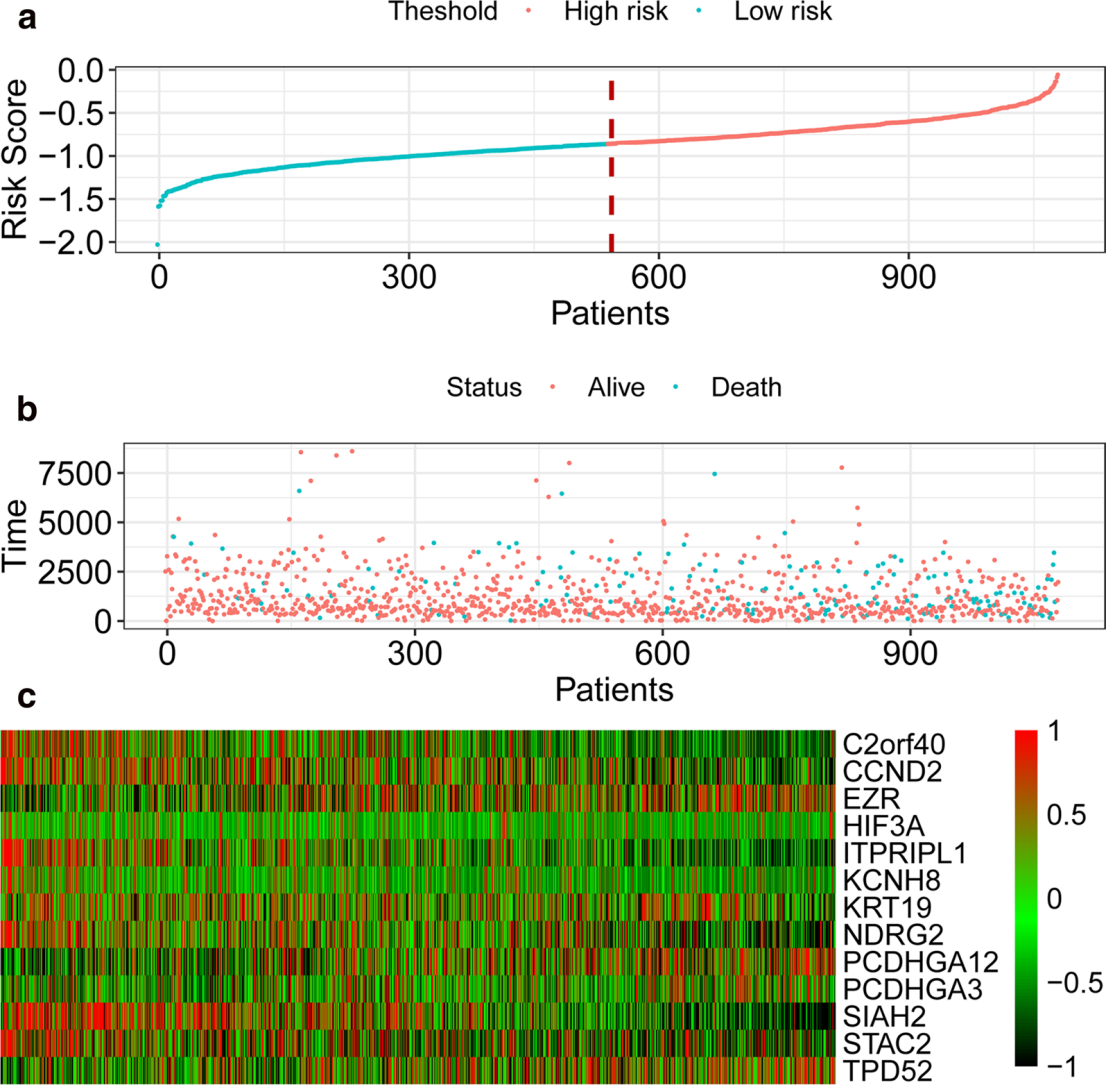
Epigenetic signature-based risk score in the training and inner validation cohort. **a** Risk score per patient. **b** Survival status. **c** Heatmap for the 13 genes

**Fig. 3:**
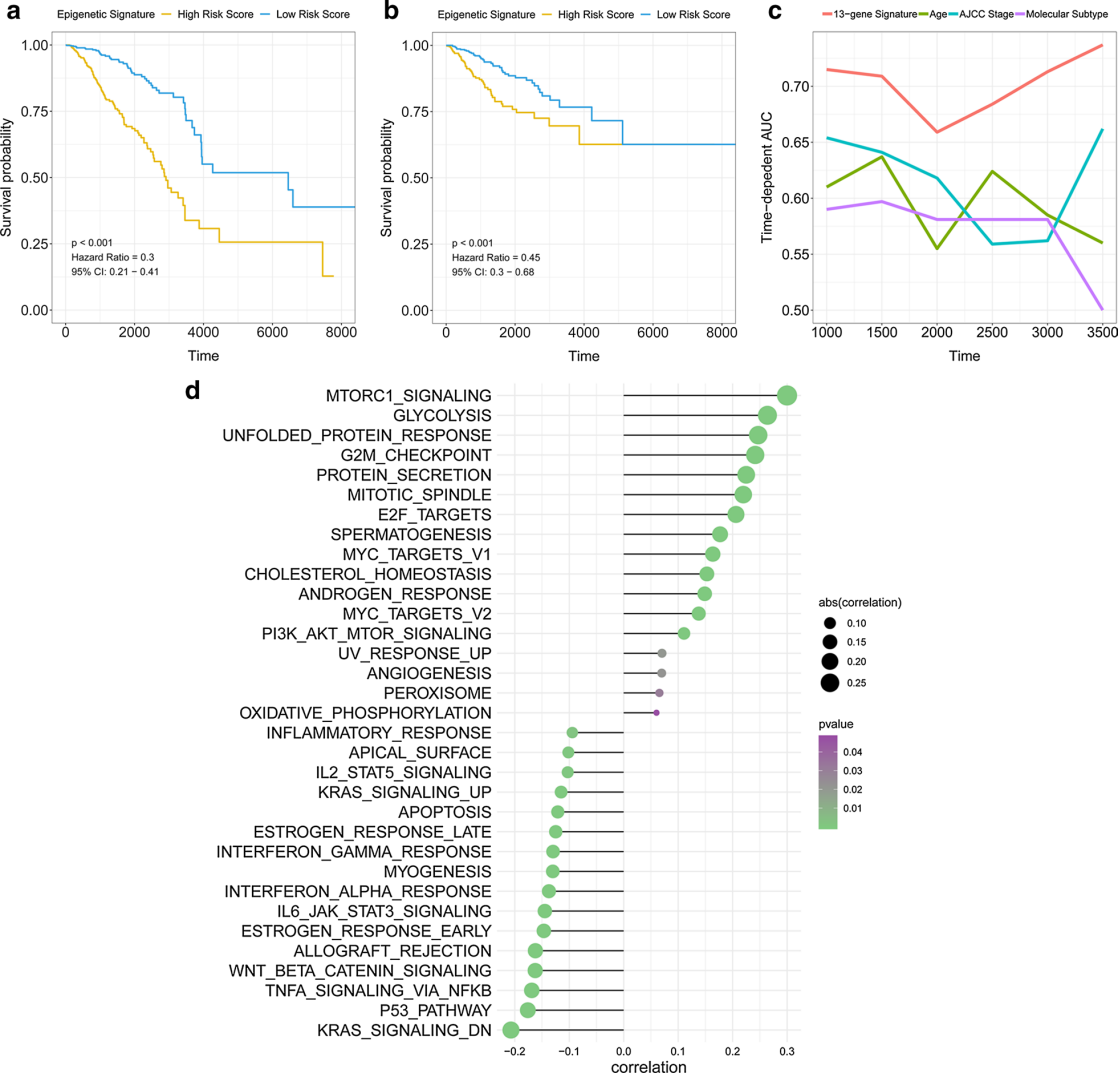
The prognostic model in breast tumour. **a** The Kaplan–Meier curve for OS in patients with breast tumour. **b** The Kaplan–Meier curve for RFS in patients with breast tumour. **c** Time-dependent ROC analysis for the epigenetic signature, TNM stage, age and molecular subtypes. **d** ssGSEA showed the correlation between the hallmarks and the epigenetic signature

A 13-gene epigenetic signature was built by the expression level of the 13 genes and the weighted parameter (formula in the method section) to predict the survival of patients with breast cancer. A median cut-off value was applied to stratify breast cancer patients into a high-risk group (n = 543) and a low-risk group (n = 544) (Fig. 2a). The survival status and heatmap for the expression of the 13 genes were showed in Fig. 2b, c. The Kaplan–Meier curve indicated patients in the low-risk group have a significantly better overall survival (OS) (HR = 0.3) and relapse-free survival (RFS) (HR = 0.45) compared to those in the high-risk group (Fig. 3a, b). The time-dependent ROC analysis revealed the 13-gene epigenetic signature had the best capacity to predict OS compared with that of other clinicopathological properties (Fig. 3c). Moreover, the correlation between the risk scores from epigenetic signature and ssGSEA scores were analysed and results showed signs of cancer-related hallmarks, e.g. mTOR signalling, G2M checkpoints, MYC targets significantly correlated with the risk scores (FDR q < 0.001) (Fig. 3d).

### WGCNA on the transcriptome of breast cancer patients

**Fig. 4:**
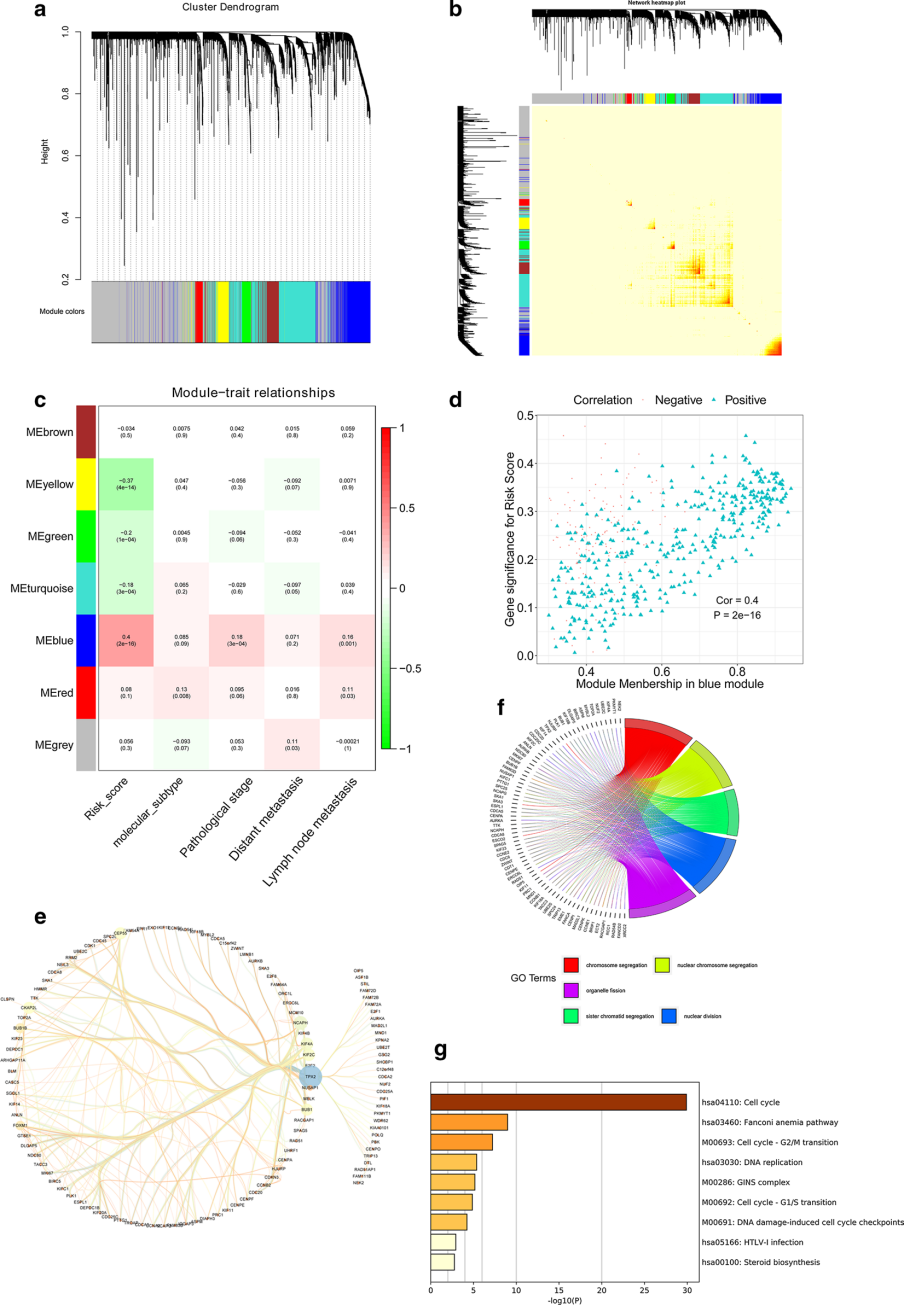
WGCNA on breast cancer RNA-seq datasets. **a** Clustering dendrogram of genes in breast cancer tissues. **b** Heatmap depicting TOM among all genes. Light colours represent low adjacency and dark colours represent high adjacency. **c** Correlation between modules and traits. **d** A scatter plot of GS for risk score versus MM in blue module, with correlation coefficient = 0.4 and p = 2e^−16^. **e** Visualisation of genes in the blue module with weights higher than the threshold (weight > 0.15). **f** GO analysis on the hub-genes. **g** KEGG analysis on the hub-genes

For a better understanding of the molecular underpinnings of the clinical characteristics of the patients we applied WGCNA on the RNA-seq data matrix. Genes from RNA-seq data matrix were applied to build a gene co-expression network (Fig. 4a). The heatmap in Fig. 4b plots the topological matrix among the transcriptome (Fig. 4b). The relationships between clinical traits (molecular subtypes, pathological stage, distant metastasis, lymph node metastasis) and the eigenvalue of each gene module are presented in Fig. 4c. The blue module, which had the highest correlation (Cor = 0.4, p = 2e^−16^) with the 13-gene signature, was selected for further analysis. The genes in blue module, which had absolute values of correlation coefficients with the 13-gene signature greater than 0.2, were identified as hub-genes. The scatterplot below illustrates the strength of the link between the 13-gene signature and the module membership for each gene in the blue module (Fig. 4c). The gene co-expression network in the blue module were analysed by cytoscape (Fig. 4d). A variety of cell cycle-related genes, such as E2F, KIF2C, CDK1 and RA7D51, were included in the network (Fig. 4e). Submitting these hub-genes to GO and KEGG analysis a strong relationship between cell division, cell cycle and 13-gene epigenetic signature is apparent (Fig. 4f, g).

### DNA methylation pattern, gene expression level in tumour and normal tissues and association of OS and RFS for the 13 genes

**Fig. 5:**
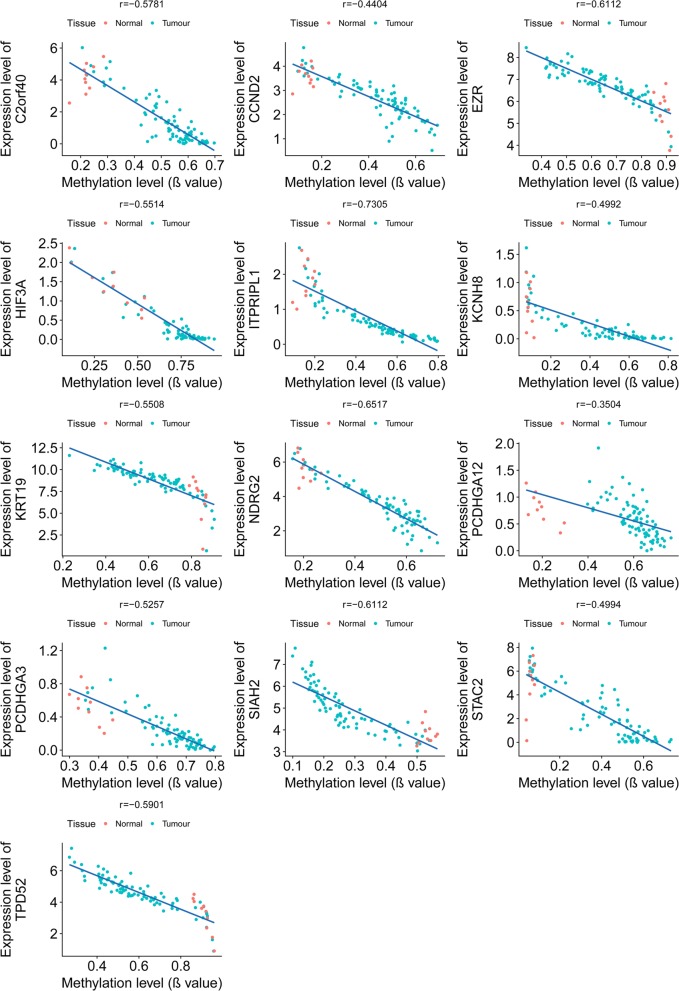
The correlation between gene expression and DNA methylation level in breast tissues

ITPRIPL1, SIAH2, KCNH8, KRT19, NDRG2, STAC2, TPD52, EZR, PCDHGA12, HIF3A, PCDHGA3, C2orf40, CCND2 were the 13 features (genes) in our LASSO Cox model. The correlation between DNA methylation status and gene expression is shown below (Fig. 5). All 13 genes show a high correlation between gene expression and DNA methylation level. The expression level of these 13 genes in different molecular subtypes of breast cancer is shown in Fig S4. Results revealed that the expression profiles were different in the 4 molecular subtypes of breast cancer. Then, the association between the expression of single gene and the OS and RFS of breast cancer patients was analysed using the Kaplan–Meier curve and univariate cox analysis (Additional file 7: Figure S5 and Additional file 8: Figure S6).

### Subgroup analysis on the 13-gene epigenetic signature

As shown in Additional file 9: Figure S7A–F, the prognostic epigenetic signature serves as a promising biomarker for predicting the survival of breast cancer in different subgroups, including Luminal A type (p = 0.03), Luminal B type (p = 0.026), HER2-enriched (p = 0.012) and triple negative (p = 0.004), stage I-II (p < 0.001), stage III-IV (p < 0.001) patients, respectively.

### Validation of the 13-gene epigenetic signature by independent breast cancer datasets

**Fig. 6:**
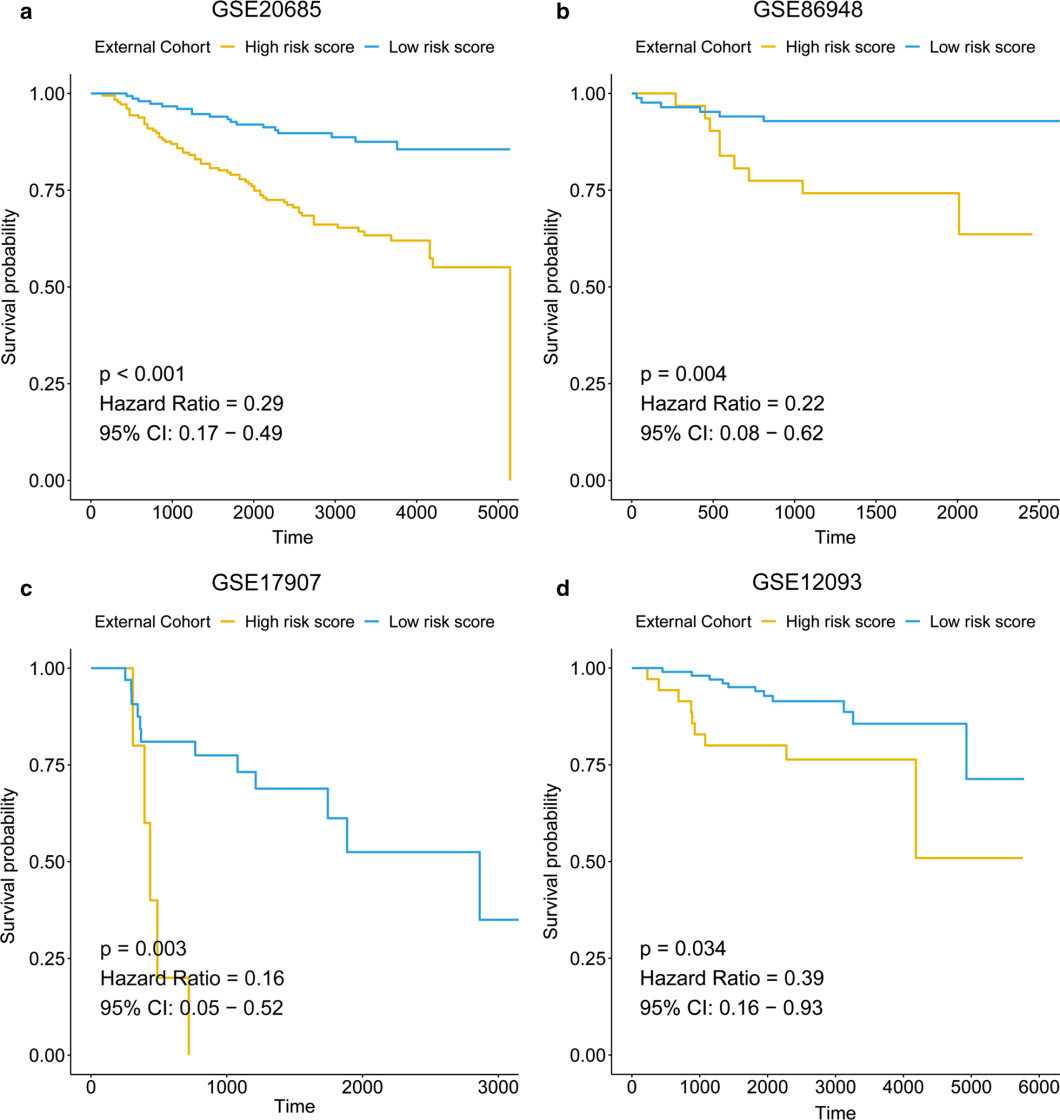
Validation of the signature in three external cohorts. Patients with a low risk score showed better OS in the validation cohorts GSE20685 (**a**), GSE86948 (**b**) and GSE17907 (**c**). Patients with a low risk score showed better RFS in the validation cohort GSE12093 (**d**)

Four independent external GEO cohorts (GSE20685, GSE86948, GSE17907 and GSE12093) (Table 1) were applied to confirm the predictive value of the 13-gene epigenetic signature. The risk score for each patient was calculated by the formula we obtained from the training set (TCGA cohort). GSE20685, GSE86948, and GSE17907 have OS as the endpoint, and GSE12093 has disease-free survival (DFS) as the endpoint. The Kaplan–Meier curve showed a significantly worse survival in the high-risk group than in the low-risk group in GSE20685 (p < 0.001) (Fig. 6a), GSE86948 (p = 0.004) (Fig. 6b), GSE17907 (p = 0.003) and GSE 12093 (p = 0.034) (Fig. 6c).

### Construction of a nomogram

**Fig. 7:**
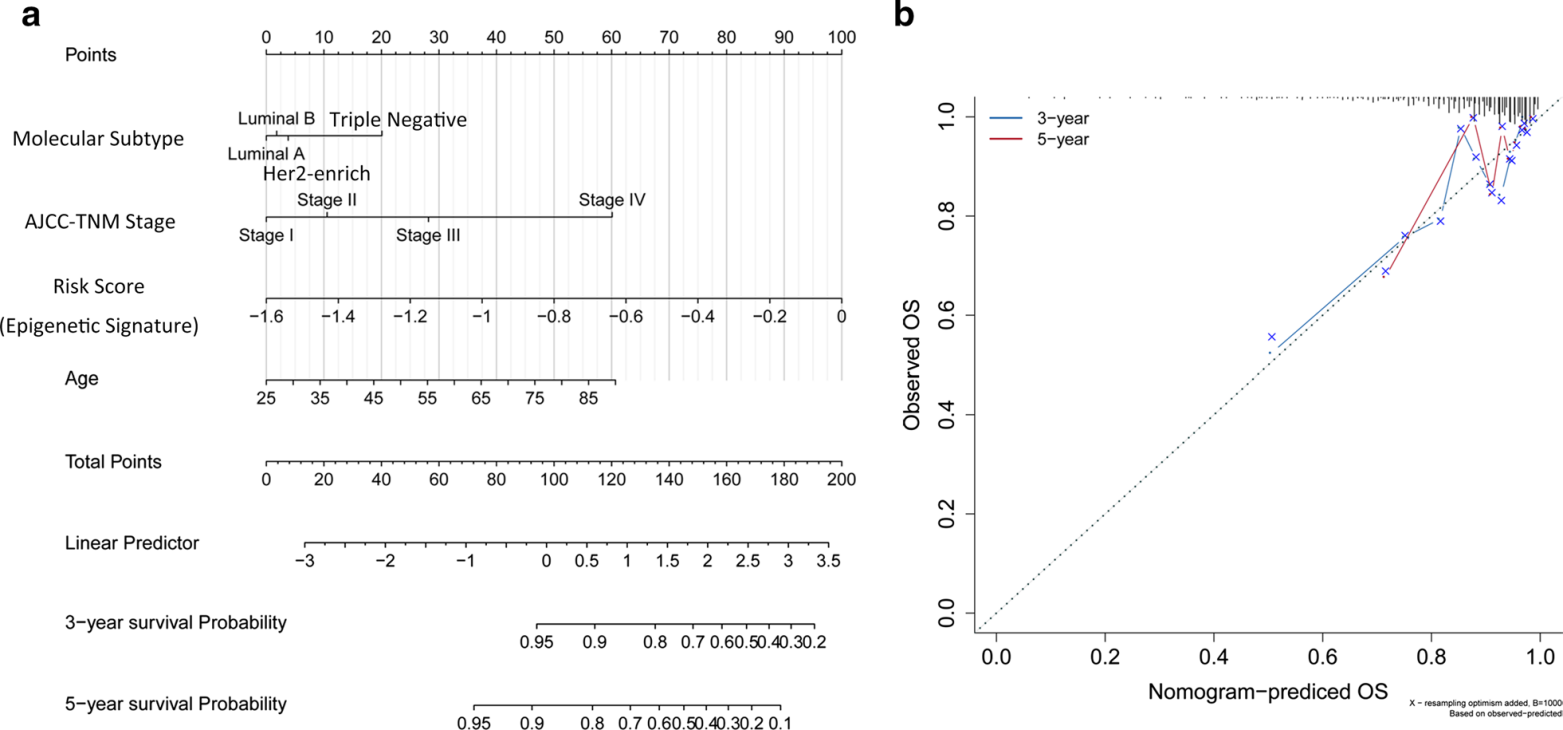
Construction of a nomogram for survival prediction. **a** Nomogram including the epigenetic signature and clinicopathological traits. **b** Calibration plot

To provide the clinician with a quantitative method by which to predict a patient’s probability of OS, a nomogram that integrated the 13-gene epigenetic signature, stage and molecular subtypes was constructed (Fig. 7a). The prediction efficiency was confirmed by the calibration plots (Fig. 7b).

## Discussion

Most of the established clinical markers for therapy response and survival of breast tumour are based on clinical traits with limited accuracy and specificity. Cellular markers of the tumour biology such as IHC positivity for estrogene receptor (ER), progesteron recepetor (PR), epidermal growth factor receptor 2 (HER-2), cytokeratin 5/6, epidermal growth factor receptor 1 (EGFR) and for cell proliferation (Ki67) are currently the gold standard for therapy stratification, but require considerable laboratory work and are prone to subjective bias. Nowadays, high-throughput data gives a comprehensive insight into the genomic, genetic and epigenetic change in patients [1, 20]. The high-throughput profiles help identify possible biomarkers for predicting the survival of patients and their reaction to therapy. Tumour tissues have a distinct DNA methylation landscape compared to adjacent normal tissues [21]. Hypermethylation of promoter CpG islands often associates with transcriptional silencing of the associated genes in breast cancer [22]. The different DNA methylation status makes it possible to use this as a potential tool in breast cancer detection and diagnosis. Here, we explore the utility of DNA methylation status and gene expression level in the prediction of survival of breast cancer patients. By integrating the DNA methylation profiles and gene expression profiles in breast tumour tissues and normal tissues, we built a 13-gene epigenetic signature. This way it was possible to predict with high confidence the CpG methylation status by measuring the mRNA expression status of 13 genes. This avoids the need of the laborious direct measurement of DNA methylation pattern, and on the other provides a robust set of biomarkers.

The subgroup analysis indicated that the epigenetic signature could stratify patients with high and low-risk scores well in different grades and in different molecular subtypes. The epigenetic prognostic model was applied together with grade and molecular subtypes to build a nomogram for predicting survival probability of patients with breast tumour. The prediction efficiency was confirmed by the calibration plot. Thus, the nomogram may help the clinics for better treatment and precision medicine in patients with breast tumour. The cellular pathway most clearly associated with the 13 gene epigenetic signature is mTORC signalling. mTOR signalling integrates both intracellular and extracellular signals and works as a central pathway in tumour progression and malignancy. Dysregulation of the PI3K/PTEN/Akt/mTORC1 pathway by gene mutations occurs in > 70% of breast tumours [23]. In ER+ breast cancers, PI3K/PTEN/Akt/mTORC1 pathway activation results in both estrogen-dependent and estrogen-independent ER activity and loss of therapy response to hormonal therapies [24]. PI3K/PTEN/Akt/mTORC1 pathway activation also results in resistance to HER2 inhibitors in HER2+ breast cancer. mTORC1 pathway inhibition helps overcome the resistance to anti-HER2 based molecular therapies [25]. Thus, hormonal therapy combined with mTORC1 blockage is a promising way for the treatment of breast tumour. The epigenetic signature showed the most significant correlation with mTORC1 signalling pathway, which may provide new strategy for the treatment of breast cancer.

A problem with the molecular profiling of the tumours as shown here is that it might be prone to intra-tumour heterogeneity. Whereas histo-morphological methods such as IHC immediately can show spacial pattern of marker expression—including focal subclones—across the tumour specimen, such an intra-tumour heterogeneity might be overseen with molecular profiles. A future direction of expression- and methylation profiling for tumour classification would therefore require a single-cell based approach.

## Conclusion

In summary, the novel 13-gene epigenetic signature serves as a promising prognostic model to predict the survival of patients with breast cancer, which may help the development of personalised and precise medicine in breast cancer field.

## Supplementary information

**Additional file 1.** Clinical informations for TCGA cohort

**Additional file 2.** Calculation procedure for lasso COX regression

**Additional file 3: Figure S1.** Flow chart of data preparation, processing, analysis and validation.

**Additional file 4: Figure S2.** ROC analysis for identifying tumour and normal tissues by the expression levels of the 13 genes.

**Additional file 5: Figure S3.** ROC analysis for identifying tumour and normal tissues by the methylation levels of the 13 genes.

**Additional file 6: Figure S4.** Expression values of the 13 genes in different subtypes of breast cancer.

**Additional file 7: Figure S5.** Kaplan–Meier survival analysis for the OS of patients with breast cancer according to the expression levels of the 13 genes.

**Additional file 9: Figure S6.** Kaplan–Meier survival analysis for the OS of patients with breast cancer according to the methylation levels of the 13 genes.

**Additional file 10: Figure S7.** Kaplan–Meier survival analysis in subgroups. (A) Luminal A subtype. (B) Luminal B subtype. (C)Triple negative subtype. (D) Her2-enriched subtype. (E) Stages I–II. (F) Stages III–IV.

## Data Availability

The datasets supporting the conclusions of this article are available in the Xena browser (https://xenabrowser.net/) repository.

## Abbreviations

LASSO: Least Absolute Shrinkage and Selection Operator
ssGSEA: single sample gene set enrichment analysis
GEO: Gene Expression Omnibus
DEGs: differentially expressed genes
DMGs: differentially methylated genes
WGCNA: Weighted Correlation Network Analysis
ROC: receiver operating characteristic
HR: hazard ratio
HER-2: epidermal growth factor receptor 2
ER: estrogene receptor
PR: progesteron recepetor
HER-2: epidermal growth factor receptor 2
EGFR: epidermal growth factor receptor 1
