# Supplementary figures and images for: A novel epigenetic signature for overall survival prediction in patients with breast cancer
Source: J Transl Med. 2019 Nov 20;17:380. doi: 10.1186/s12967-019-2126-6 (PMC6889649; doi:10.1186/s12967-019-2126-6)

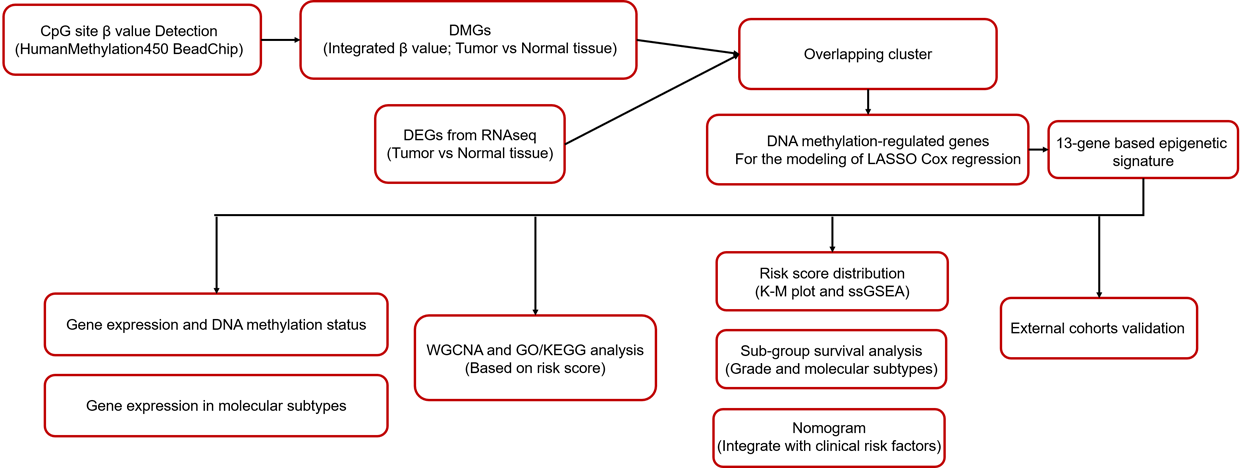

Supplement: Supplementary file 3 — Additional file 3: Figure S1. Flow chart of data preparation, processing, analysis and validation. [file 12967_2019_2126_MOESM3_ESM.tif]

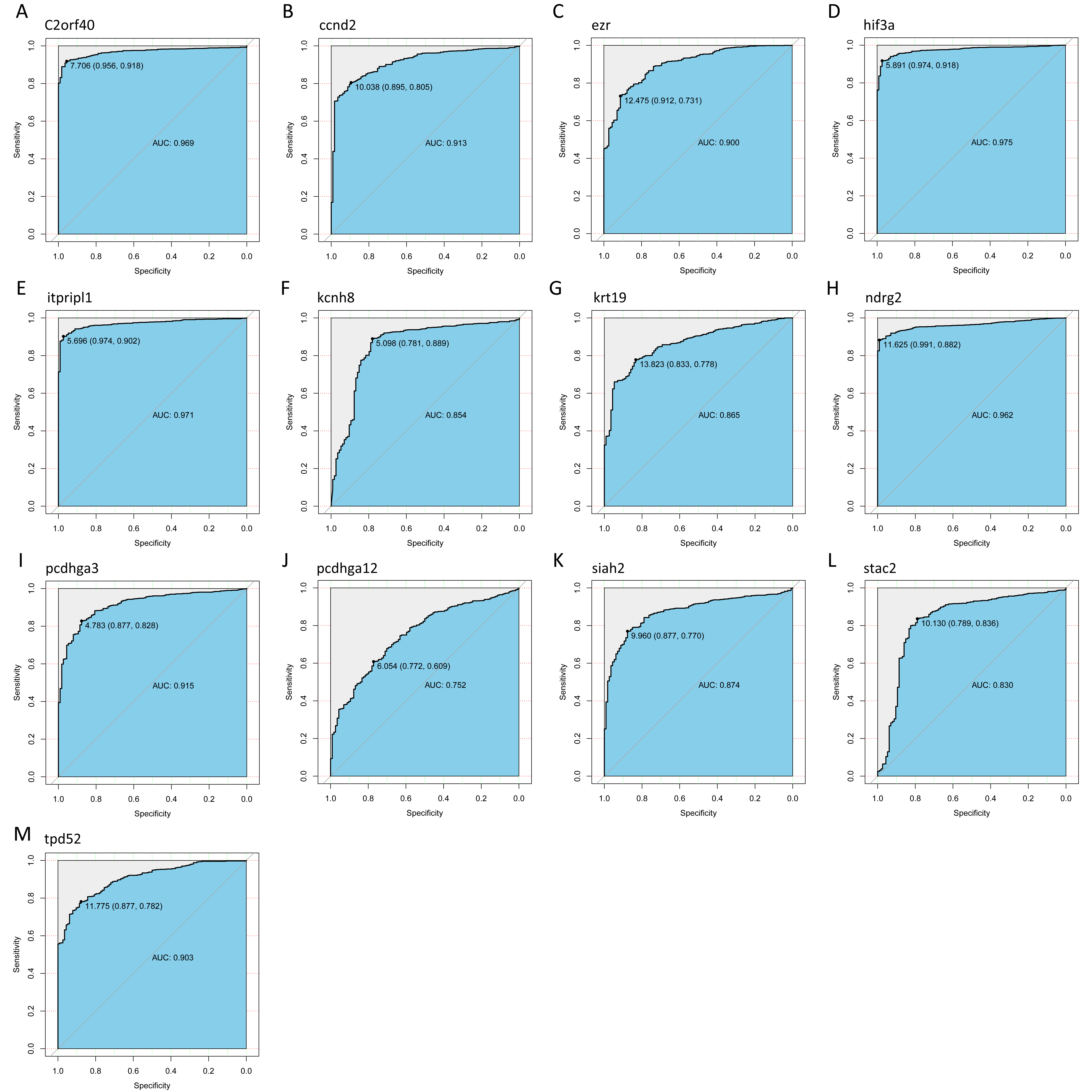

Supplement: Supplementary file 4 — Additional file 4: Figure S2. ROC analysis for identifying tumour and normal tissues by the expression levels of the 13 genes. [file 12967_2019_2126_MOESM4_ESM.tif]

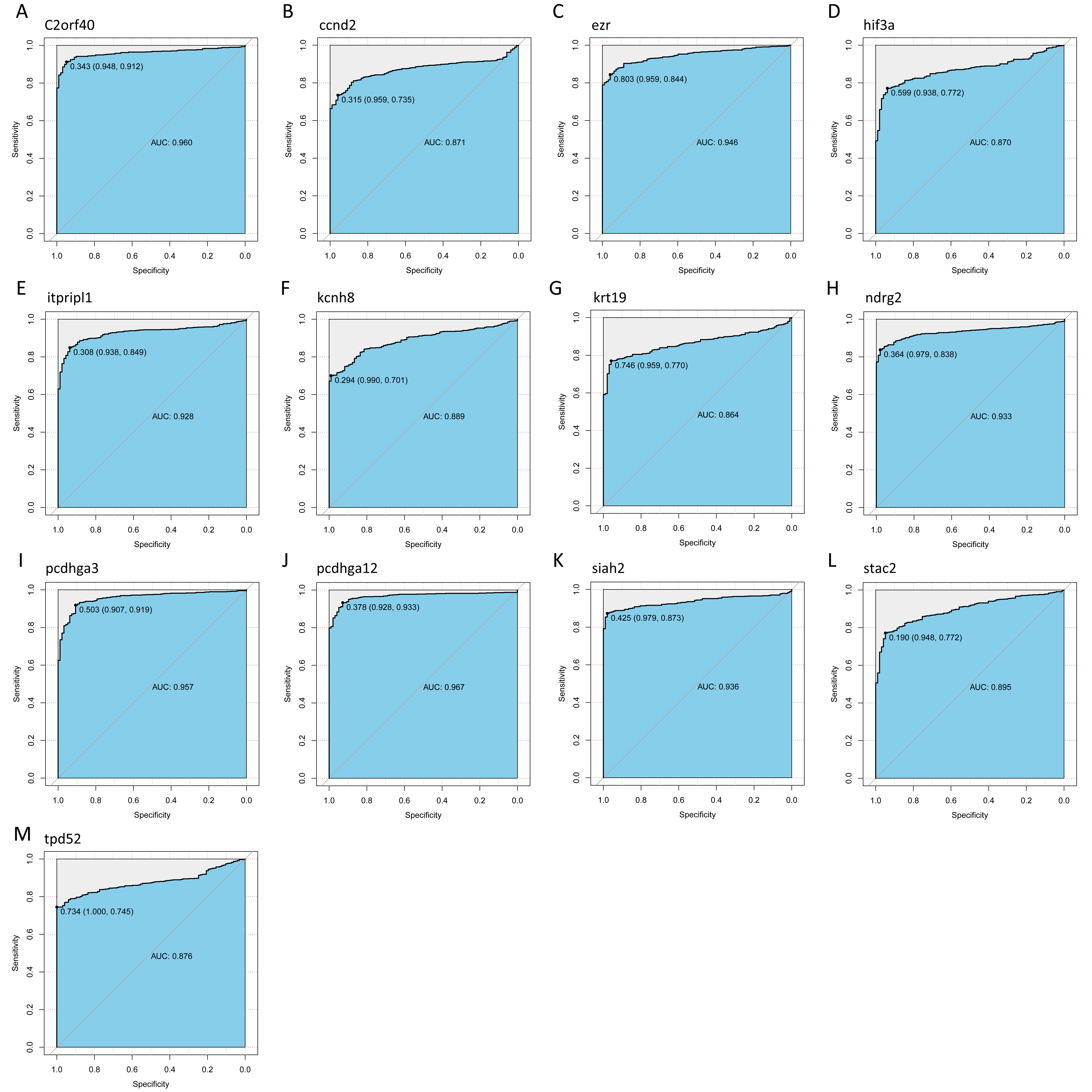

Supplement: Supplementary file 5 — Additional file 5: Figure S3. ROC analysis for identifying tumour and normal tissues by the methylation levels of the 13 genes. [file 12967_2019_2126_MOESM5_ESM.tif]

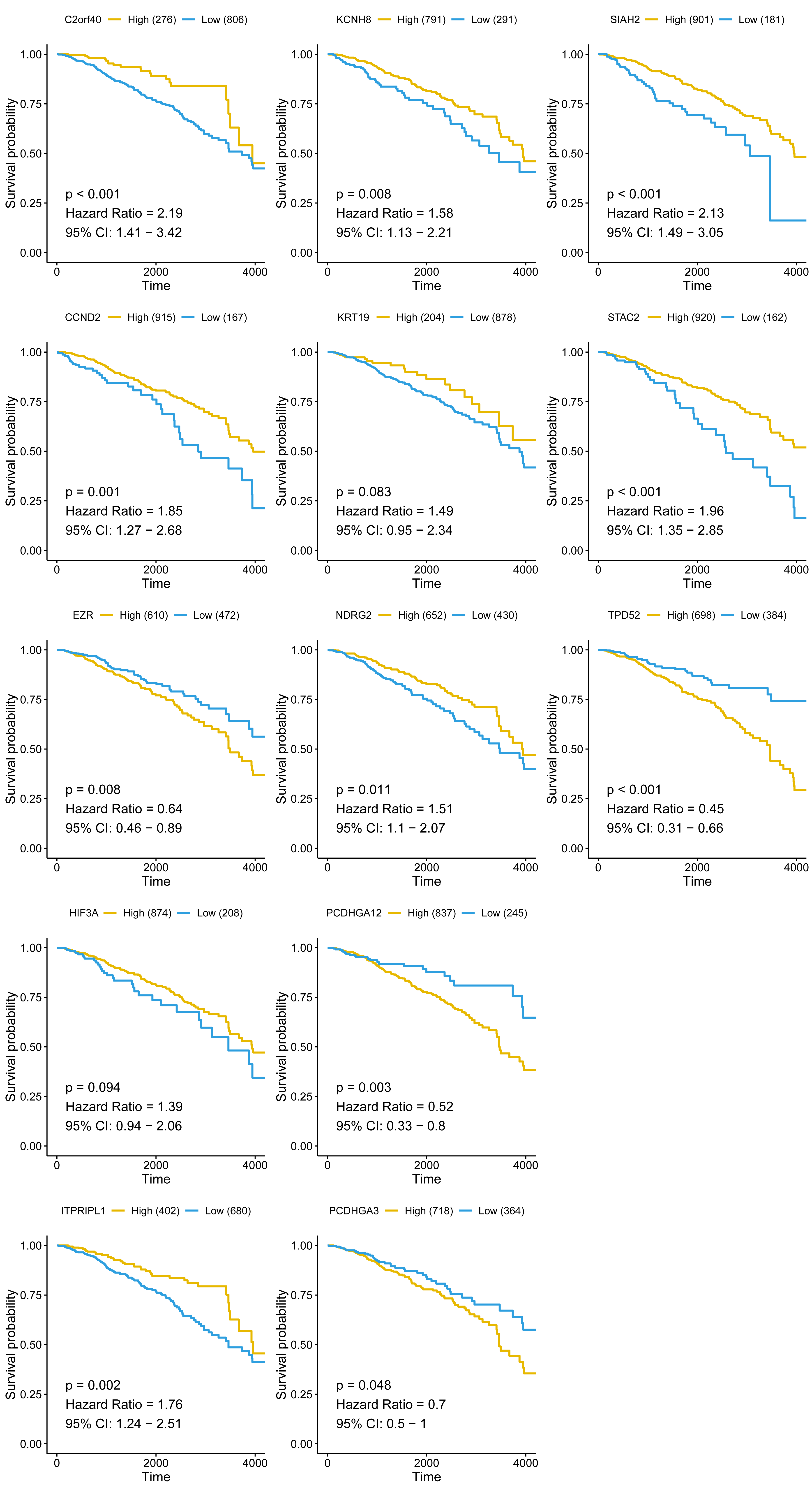

Supplement: Supplementary file 6 — Additional file 6: Figure S4. Expression values of the 13 genes in different subtypes of breast cancer. [file 12967_2019_2126_MOESM6_ESM.tif]

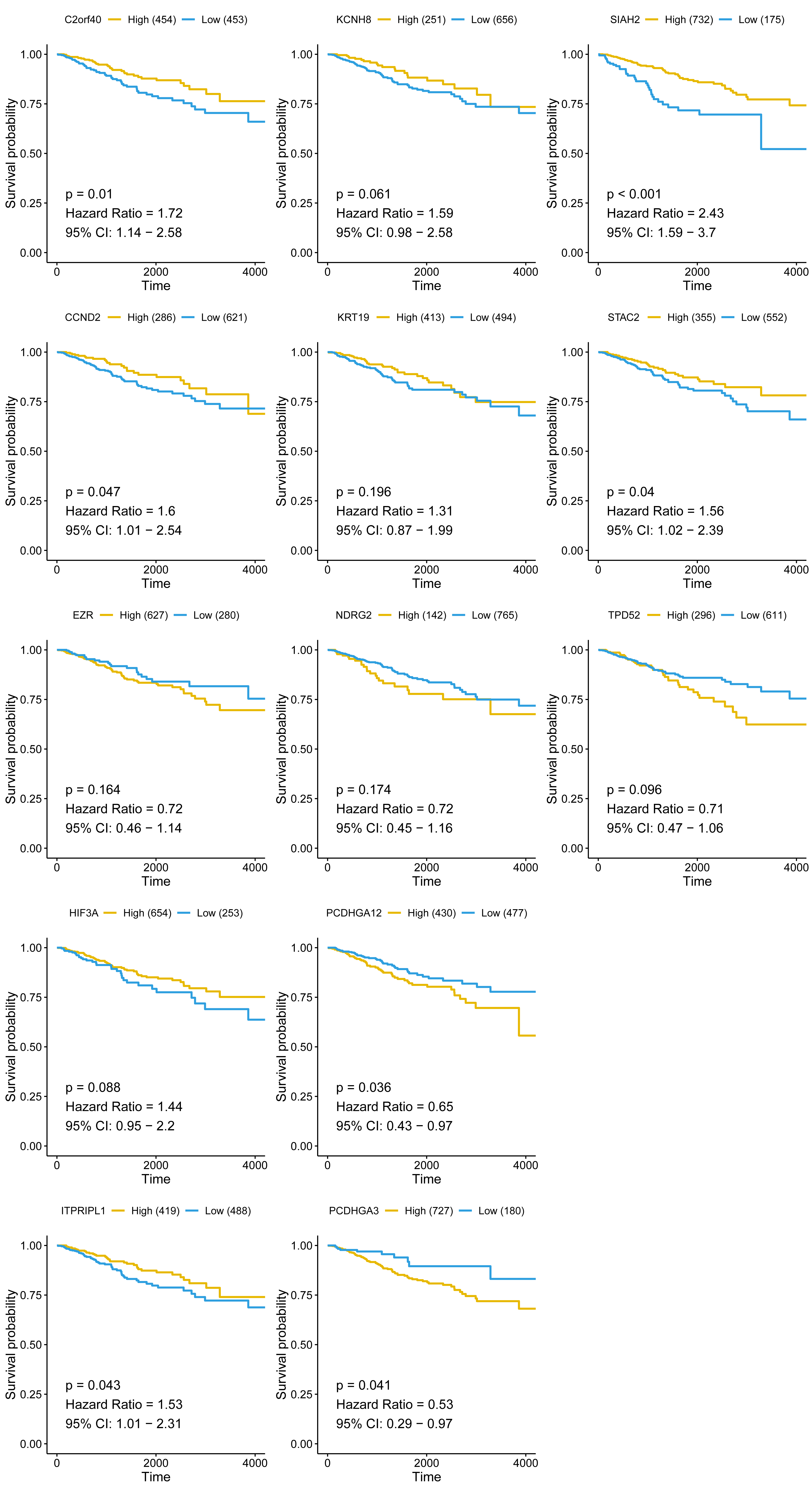

Supplement: Supplementary file 7 — Additional file 7: Figure S5. Kaplan–Meier survival analysis for the OS of patients with breast cancer according to the expression levels of the 13 genes. [file 12967_2019_2126_MOESM7_ESM.tif]

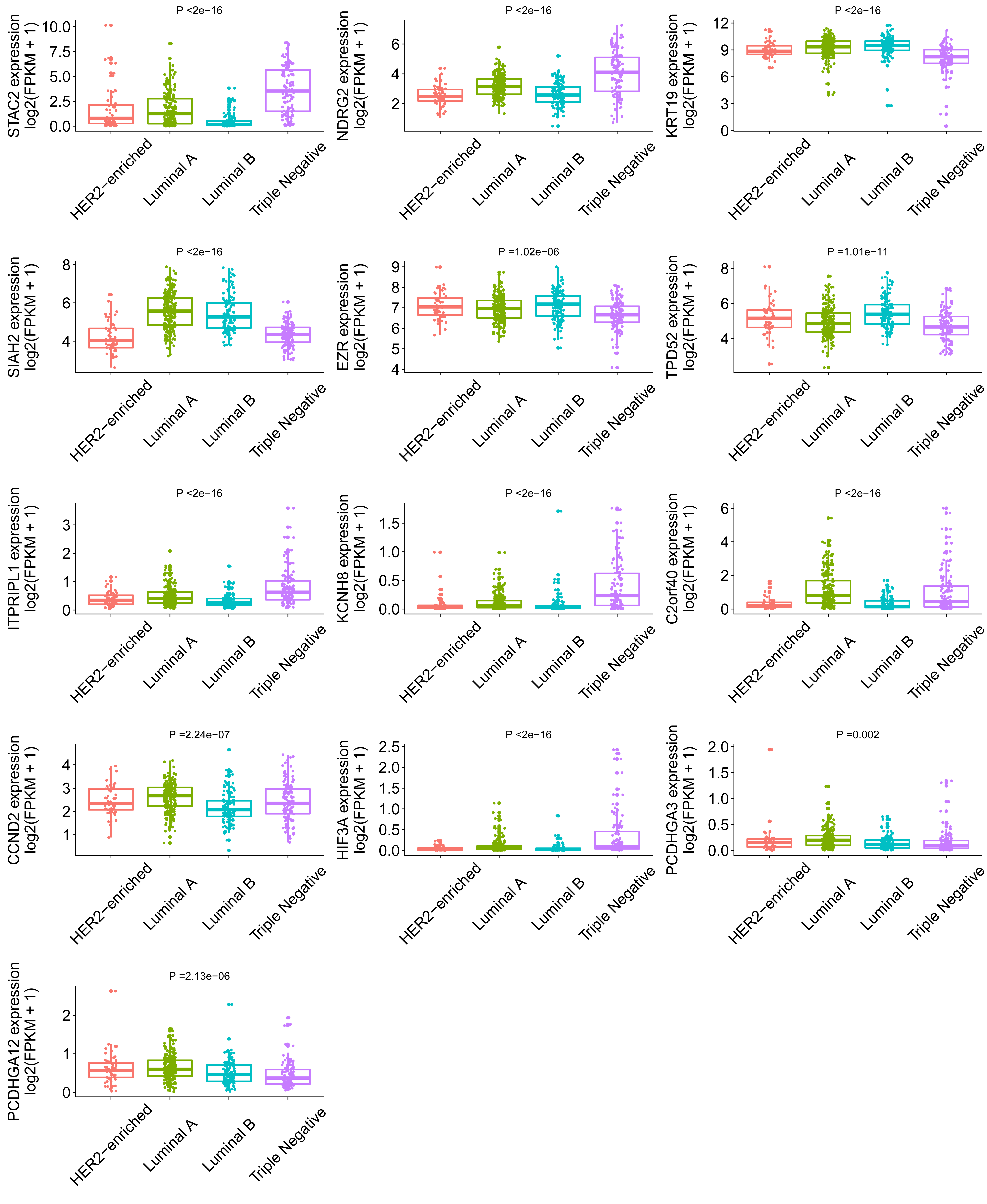

Supplement: Supplementary file 8 — Additional file 9: Figure S6. Kaplan–Meier survival analysis for the OS of patients with breast cancer according to the methylation levels of the 13 genes. [file 12967_2019_2126_MOESM8_ESM.tif]

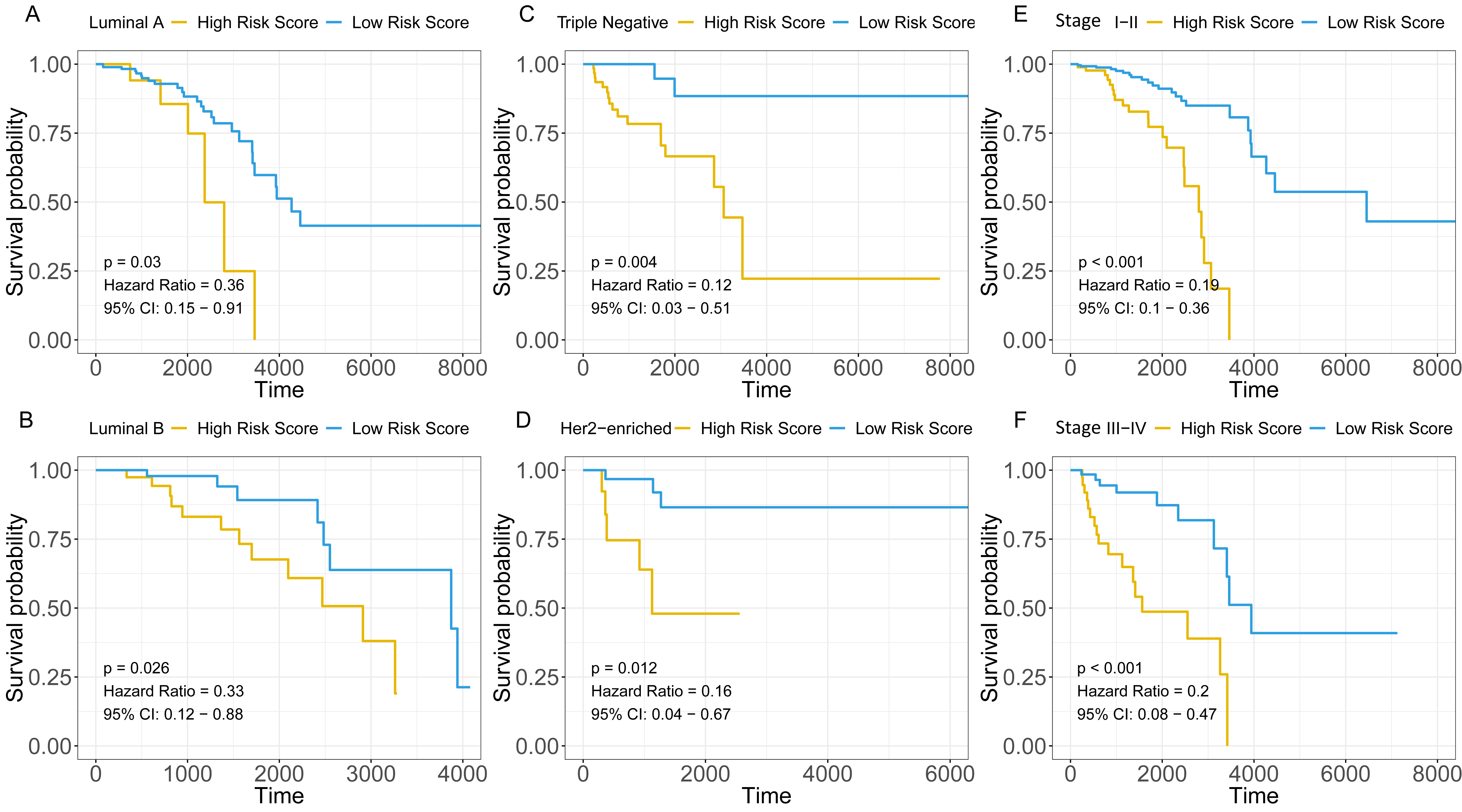

Supplement: Supplementary file 9 — Additional file 10: Figure S7. Kaplan–Meier survival analysis in subgroups. (A) Luminal A subtype. (B) Luminal B subtype. (C)Triple negative subtype. (D) Her2-enriched subtype. (E) Stages I–II. (F) Stages III–IV. [file 12967_2019_2126_MOESM9_ESM.tif]
